# EPIDEMIOLOGY OF FALLS AMONG OLDER ADULTS IN THE COMMUNITY FROM THE HEALTH AND RETIREMENT STUDY

**DOI:** 10.1093/geroni/igad104.3780

**Published:** 2023-12-21

**Authors:** Lien Quach, Elizabeth Dugan, Uyen-Sa Nguyen, Jeffrey Burr

**Affiliations:** Massachusetts General Hospital, Boston, Massachusetts, United States; University of Massachusetts, Boston, Boston, Massachusetts, United States; University of North Texas Health Science Center School of Public Health, Fort Worth, Texas, United States; University of North Texas Health Science Center School of Public Health, Fort Worth, Texas, United States

## Abstract

Many older adults experience falls that lead to adverse health consequences. The primary objective of this study is to estimate the associations between risk factors of falls and actual falls. The second objective is to identify whether race modifies these relationships. Panel data from the Health and Retirement Study from 2006-2012 were analyzed. The total sample size of community-dwelling adults age 65+ was 22,153, and respondents were observed at different time points. Independent variables included physiological characteristics and health, lifestyle indicators, social environment, and socio-economic status. At each time point, the outcome was the number of falls reported in the two years prior to the interview. Generalized Estimating Equations for negative binomial regression models adjusted for repeated measures were performed. The mean age of participants in 2006 was 74, and 38% experienced at least one fall every two years. For each additional chronic health condition, the fall risk increased by 13%. Vision impairment increased fall risk by 21%. Conversely, higher cognitive function was correlated with a 3% reduction in fall risk. Every unit increase in ADL limitations escalated fall risk by 17%. Loneliness increased fall risk by 12%. Both vigorous (12%) and moderate (13%) physical activities were linked to a decrease in fall risk. Notably, minority racial group status modified the interplay between loneliness and falls and also bolstered the relationship between vision impairment and fall frequency. Tailoring fall prevention programs to racial and ethnic minority older adults is essential to reduce health disparities at the population level.

